# Rupture sous-cutanée du tendon long extenseur du pouce: à propos de 5 cas

**DOI:** 10.11604/pamj.2014.17.285.3035

**Published:** 2014-04-15

**Authors:** Rachid Abdelillah, Najib Abbassi, Moncef Erraji, Najib Abdeljawad, Hicham Yacoubi, Abdelkrim Daoudi

**Affiliations:** 1 Service de chirurgie orthopédique et traumatologique, centre hospitalier d’Oujda, Oujda, Maroc

**Keywords:** Tendon, extenseur du pouce, Rupture sous-cutanée, Tendon, extensor of the thumb, Subcutaneous rupture

## Abstract

La rupture spontanée du muscle long extenseur du pouce (EPL) du tendon au niveau du poignet est rare et principalement rapportés après fracture du radius distal à tubercule de Lister, dans la synovite, ténosynovite ou la polyarthrite rhumatoïde. Nous rapportons 5 cas de rupture spontanée du tendon long extenseur du pouce, traités par une greffe ou un transfert tendineux.

## Introduction

C est une affection rare qui se défini par un déficit de l extension active (rétro pulsion) de l inter-phalangienne du pouce, sans ouverture cutanée. Il complique souvent une fracture de l extrémité inférieure du radius, une ténosynovite ou une polyarthrite rhumatoïde [1]. Le traitement est chirurgical tenant compte du siège et de l ancienneté de la lésion, il se base essentiellement sur la greffe tendineuse, et le transfert du tendon extenseur propre de l index.

## Méthodes

Nous rapportons une étude rétrospective de 5 cas de rupture sous-cutanée du tendon long extenseur du pouce, traités soit par une greffe tendineuse, ou un transfert tendineux de l extenseur propre de l index.

## Résultats

**Figure 1:**
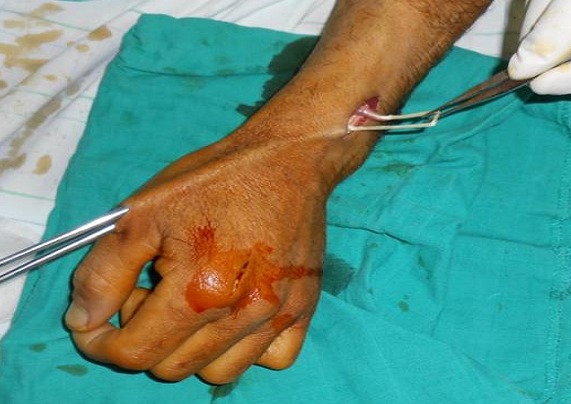
Transfert tendineux de l extenseur propre de l index

**Figure 2:**
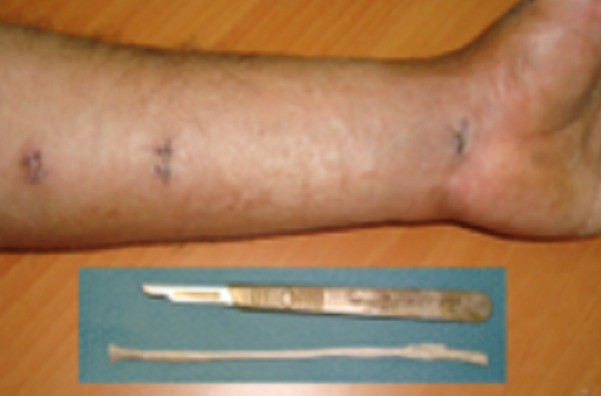
Greffe tendineuse par le tendon du muscle long palmaire

**Figure 3:**
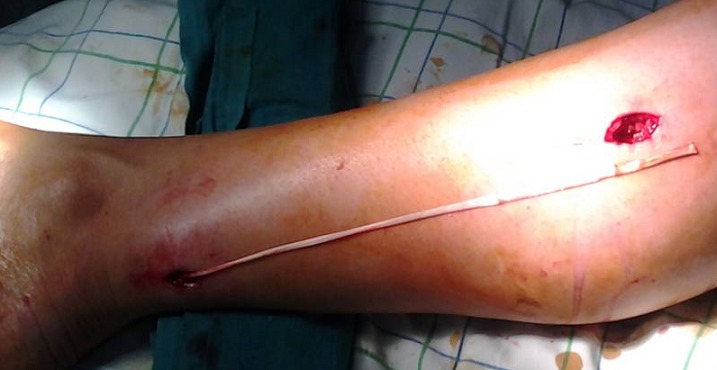
Greffe tendineuse par le tendon du muscle plantaire grêle

**Figure 4:**
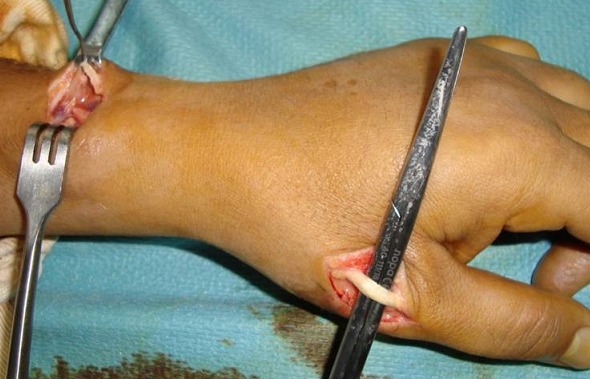
Suture tendineuse

L´âge moyen dans notre série était de 35 ans, avec un délai entre la rupture et la reconstruction qui varie entre 4 semaines et 2 ans. L étiologie était une fracture de l extrémité inférieure du radius non déplacée dans 2 cas, une polyarthrite rhumatoïde chez 1 patient et un microtraumatisme répétitif chez 2 patients. La suture termino-terminale était impossible pour tous les cas, faisant appel à un transfert du tendon extenseur propre du 2ème doigt dans 1 cas (Figure 1), où le bout proximal du tendon était introuvable, et une greffe tendineuse dans 4 cas, en utilisant le tendon du muscle long palmaire chez 3 patients (Figure 2), et le tendon du muscle plantaire grêle chez un seul patient qui n avait pas du muscle long palmaire (Figure 3). La suture tendineuse s est faite en regard de la face dorsale de l articulation métacarpo-phalangienne en pulvertaft (Figure 4). La tension du tendon a été vérifié par l effet ténodèse du poignet, avec une possibilité de fléchir l interphalangienne du pouce sur un poignet en extension. Tous nos patients ont eu une immobilisation, pouce en abduction, pendant 4 semaines, suivi d une rééducation activo-passive jusqu´à la récupération des amplitudes normales du pouce.

**Figure 5:**
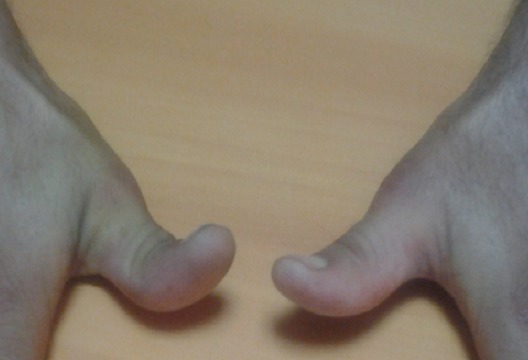
Résultats clinique

Après un recul moyen de 1 an tous nos patient ont bien évolué sur le plan fonctionnel avec une opposition selon Kapandji de 10/10, et une extension complète de l interphalangienne du pouce (Table 1, Figure 5).

**Table 1:** Résultats fonctionnel en fonction de la technique chirurgicale

	Score de Kapandji	Rétropulsion (par rapport au côté sain)
Extenseur propre de l’index	10/10	80%
Long palmaire	10/10	90%
Plantaire grêle	10/10	90%

## Discussion

La rupture sous cutanée du tendon long extenseur propre du pouce reste la principale complication à craindre dans les fracture de l extrémité inferieure du radius ; incidence estimée à 5% [1].

D autres causes peuvent être à l origine de la rupture: La prise des corticoïdes; Bjorkman et Jorgsholm [2] ont rapporté 2 cas de rupture du LEP juste après injection de cortisone; les microtraumatismes répétitifs, Chul han kim [3] rapporte un cas de rupture du LEP dans le cadre d une activité professionnelle, chez une cuisinière, traitée par un transfert tendineux de l extenseur propre du 2ème doigt; la polyarthrite rhumatoïde: soit en rapport avec la déformation osseuse ou l inflammation de la synoviale [4]. Zvijac et al. [5] ont rapporté que le tendon EPL pourrait spontanément exploser sous l´effet de l´attrition du tendon autour du tubercule de Lister.

La physiopathologie de cette rupture du LEP peut être expliquée par 2 théories : une théorie mécanique, en rapport avec le contact étroit du LEP et le tubercule de lister, siège fréquent des fractures de l extrémité inferieure du radius [6], une théorie vasculaire, qui met en relation direct le siège de la rupture du tendon et sa vascularisation précaire, confirmée par des études microangiographiques [7].

Le traitement était toujours chirurgical. Plusieurs techniques chirurgicales ont été pratiquées au début (8), à savoir la suture du bout distal du tendon avec les tendons court extenseur et long abducteur du pouce, ou avec le tendon extenseur ulnaire du carpe, toutes ces techniques n arriveraient pas à restaurer l extension de l inter-phalangienne du pouce. Actuellement, il existe deux principales techniques pour restaurer la fonction du tendon du muscle long extenseur du pouce à savoir la greffe tendineuse utilisant le plus souvent le tendon du muscle long palmaire, et le transfert du tendon extenseur propre de l index qui reste la plus décrite dans la littérature, T. Apard [1] a adopté la même technique pour traiter une rupture du LEP sur rhizarthrose très évoluée.

L analyse de nos résultats se concorde avec les données de la littérature (Table 2).

**Table 2:** Comparaison avec les données de la littérature

	Très bonne	Bonne	Modéré	Mauvais
Saur M. et al. [8], Nombre = 48	10%	73%	17%	**-**
Ozalp T. et al. [9] Nombre = 25	56%	24%	16%	4%
Notre série, nombre = 5	40%	60%	0	**-**

## Conclusion

Les ruptures sous cutanées du long extenseur du pouce sont relativement rares, où le diagnostic est basé sur la clinique, plusieurs étiologies peuvent être impliquées, alors que le traitement est chirurgical basé sur le transfert ou la greffe tendineuse.
